# Evaluation of a Novel Simulation Curriculum With the Segmented Model in Pediatric Cardiovascular Education

**DOI:** 10.3389/fpubh.2022.887405

**Published:** 2022-05-20

**Authors:** Ying Yang, Lan-Fang Tang, Chun-Zhen Hua, Jian-Hua Mao, Yun-Xia Hong

**Affiliations:** ^1^Department of Infectious Diseases, The Children's Hospital, Zhejiang University School of Medicine, National Clinical Research Center for Child Health, Hangzhou, China; ^2^Respiratory Department, The Children's Hospital, Zhejiang University School of Medicine, National Clinical Research Center for Child Health, Hangzhou, China; ^3^Nephrology Department, The Children's Hospital, Zhejiang University School of Medicine, National Clinical Research Center for Child Health, Hangzhou, China; ^4^Education Office, The Children's Hospital, Zhejiang University School of Medicine, National Clinical Research Center for Child Health, Hangzhou, China

**Keywords:** pediatric cardiovascular specialty, simulation education, residency training, novel model, teaching effectiveness

## Abstract

**Objective:**

The need to develop the full range of knowledge, skills, and professionalism poses new challenges for pediatric cardiovascular education. This study aimed to investigate the effectiveness of a novel simulation curriculum with the segmented model for pediatric cardiovascular residents.

**Materials and Methods:**

First, the simulation course was designed according to a prior survey and based on a human patient simulator setting. Then, another 55 residents were randomly selected and assigned to participate in a simulation course (about acute fulminant myocarditis in children), either in the experimental group or the control group. Taking full advantage of the simulation education, the simulation case in the experimental group was divided into three segments and included a micro-debriefing at the end of each segment. The three segments were independent but together formed the whole case. It was designed through three cycles of running and debriefing, and more challenging tasks were gradually proposed to residents. The case in the control group was not split and included only one case running and debriefing. The assessments of the residents' knowledge, skills, professionalism performance, and satisfaction feedback from residents were analyzed to evaluate the effectiveness of the course.

**Results:**

In total, 44 residents completed the whole experimental period, including 23 participants in the experimental group and 21 participants in the control group. The pre-course knowledge assessment scores of the two groups were comparable, while the mean post-course score in the experimental group was 82.61 ± 17.38, which was significantly higher than that in the control group (50.48 ± 18.57, *p* < 0.01). The mean skills assessment score of residents in the experimental group was 84.17 ± 6.01, which was significantly higher than the control group (54.50 ± 5.72, *p* < 0.01). In terms of the professionalism assessment, the residents in the experimental group achieved better performance than those in the control group in all aspects (respect, privacy, communication, responsibility, and cooperation) (*p* < 0.05). Satisfaction feedback from residents showed that self-confidence regarding knowledge mastery in the experimental group was significantly higher than that in the control group (*p* < 0.05), while there were no significant differences in the evaluations of the teacher's performance (*p* > 0.05).

**Conclusions:**

The novel simulation curriculum with the segmented model helps residents achieve better performance in terms of knowledge, skills, and professionalism while improving self-confidence. It has some value in pediatric cardiovascular education and is worthy of further promotion.

## Introduction

There is an irreversible trend of pediatric cardiovascular doctors needing to provide high-quality care to children, which means that developing a full range of knowledge, skills, and professionalism is necessary (1). Deficiencies in any one of these aspects will dramatically affect the outcomes for pediatric cardiovascular patients. However, in some developing countries, reports from both teachers and residents show that there is a big gap between goals and reality. The mastery, retention, and application of knowledge and skills among residents are lower compared to senior doctors due to their relatively short clinical working time. The cultivation of professionalism depends more on personal contact with the clinical environment. The relatively low level of knowledge and skills among residents leads to their lack of self-confidence in dealing with clinical problems, which further limits their abilities in all aspects. The need for an effective educational model that can simulate the real medical environment with high-fidelity is urgent. In recent years, simulation-based medical education (SBME) has been applied to pediatric teaching to improve this situation in some developed regions of China and was reported to be effective (2). SBME uses simulators or humans as substitutes for real patients and provides an educational environment for educators to use—a real but well-controlled clinical experience, simulating real-life patient care (3). It was reported to be well suited for both high-risk and low-volume clinical scenarios, such as pediatric cardiovascular medicine (3). The knowledge and skills required in these scenarios are rarely practiced during clinical care and can instead be practiced frequently in simulation scenarios without endangering patients. The key to a simulation curriculum is the integration of the most compatible model, which has been a major challenge for pediatric SBME for the past 20 years. A compatible model will improve the effectiveness of the simulation curriculum, while an inappropriate teaching model will result in a waste of time and resources. The two main features of the SBME facility's effective teaching are providing debriefings and repeated practices, according to the findings of a recent system analysis (4). Debriefings are fairly important, and various studies have shown that they can improve learning efficiency and memory retention in SBME (5, 6). SBME with repeated practices is better than those without them in skills acquisition (7). Our study tried to combine the two merits of SBME in a novel model to make full use of them.

Acute fulminant myocarditis (AFM) is rare inflammatory heart disease. The overall incidence of acute myocarditis was 1.4–2.1 per 100,000 children, and 22.8–44.1% of acute myocarditis was classified as AFM, according to a study from 2007 to 2016 conducted in Korea and a study from 2012 to 2018 conducted in Japan (8, 9). However, a significant increase has been found in the incidence rate of AFM over time (8). In particular, AFM in children has been reported to lead to a higher mortality rate than in adults (10). A retrospective observational study of children showed that the mortality related to AFM was 24.1%, which is 41.3 times higher than that of non-fulminant myocarditis (9). AFM has a sudden onset and rapidly develops into heart failure, cardiogenic shock, fatal arrhythmia, or multi-organ failure. Its early symptoms are non-specific, including abdominal pain, vomiting, fatigue, syncope, and convulsions (11), often leading to misdiagnoses (12). The targeted history collection, physical examination, and laboratory tests and imaging (myocardial injury biomarkers, electrocardiogram, echocardiography, cardiac magnetic resonance, etc.) are helpful for early identification and differential diagnosis (11). The management strategy of AFM is to maintain stable hemodynamics and adequate organ perfusion. Even with active medications (intravenous immunoglobulin or glucocorticoids) and supportive therapy (fluid resuscitation or anti-arrhythmic treatment), some patients will still present lactic acidosis and low peripheral perfusion. The only way to improve the survival rate is to maintain the children's circulation with extracorporeal membrane oxygenation (ECMO). According to data from the Extracorporeal Life Support Organization in 2018, AFM had the highest survival rate among all disease types supported by ECMO (up to 76%) and a short hospitalization duration (8 days). The long-term cardiac function of these children during follow-up was satisfactory (13).

We can see that AFM is a disease that is easily misdiagnosed. Early diagnosis and targeted therapy will, thus, significantly improve patients' prognoses. Appropriate management of a child with AFM requires doctors to have related knowledge, skills, and professionalism (communication, cooperation, and so on). However, the chance for residents to understand AFM in clinical practice is very low due to its low incidence rate. Therefore, taking AFM in children as an example, our team developed a novel simulation course with the segmented model characterized by providing debriefings and repeated practices and applied it to the education of residents. The main aim was to build a bridge between pediatric cardiovascular residents and clinical diseases.

## Materials and Methods

### Simulation Course Design

A survey of the simulation course about AFM in children was conducted. Ten third-grade pediatric cardiovascular residents in the Children's Hospital of Zhejiang University Hospital (Hangzhou, China) were randomly selected from May 2021 to June 2021 to complete the survey. Contents chosen by over 70% of residents were included in the course according to the survey. The course was designed based on a human patient simulator setting (PediaSim ECS from Tellyes Scientific Company, Tianjin, Hebei, China) according to the results of the survey. Taking full advantage of the simulation education, the simulation case in the experimental group was divided into three segments and included a micro-debriefing at the end of each segment. The three segments were independent but related to each other and arranged in order, together forming the whole case. It was designed through three cycles of running and debriefing, and more challenging tasks were gradually proposed. The case in the control group was not split, and the main content was continuously displayed, including only one case running and debriefing.

### Simulation Course Practice

Another 55 third-grade pediatric cardiovascular residents from the Children's Hospital of Zhejiang University School of Medicine were randomly selected to participate in the simulation course practice. Participants were subsequently assigned into two groups randomly: the experimental and the control groups.

Participants were scheduled to spend 8 weeks completing the experimental period. On the first day of the first week, they were given the same preview materials (including textbooks, clinical guidelines, expert consensus, pictures, videos, etc.). One-to-one supervision was conducted to ensure that all residents had the same preview time before the course. On the first day of the second week, both groups of residents were scheduled to complete the pre-course test. In the control group, a 48-min simulation course practice was conducted on the first day of the third week to complete the whole case. In the experimental group, a 16-min simulation course practice was conducted on the first day of each week from the third to the fifth week to complete the three segments of the case, respectively. The same teacher guided the practice in both groups. The residents participated in the course practice by role-playing as doctors or nurses. Every five residents were assigned to a team, playing the role of a junior cardiovascular pediatrician, a senior cardiovascular pediatrician, an ECMO physician, and two cardiovascular nurses, respectively. During this period, the two groups of residents were given clinical practice and bedside teaching of no difference. On the first day of the ninth week, both groups of residents underwent the post-course test, followed by satisfaction feedback.

### Effectiveness Evaluation

The evaluation of the simulation course's effectiveness included three parts: knowledge, skills, and professionalism assessment. The knowledge tests were carried out before and after the course, randomly selected from the same examination question bank. Skills and professionalism assessments were conducted after the course. Both knowledge and skills were assessed by scores with the highest possible score being 100. Professionalism was assessed by level: dissatisfied, average, and satisfied. The satisfaction feedback from residents included two parts: their own performance and that of the teachers, ranked by level: very dissatisfied, dissatisfied, average, satisfied, and very satisfied. The data from any participant who didn't complete the whole simulation course practice, the entire knowledge, skills, and professionalism assessment, or the satisfaction feedback portion, were excluded while evaluating the simulation course's effectiveness.

### Statistical Analysis

Categorical data were expressed as numbers and percentages (*n*, %) and were compared using the χ^2^-test between groups. Continuous data with normal distribution were expressed as X ± S and were compared with a *t-*test. Continuous data with a non-normal distribution were expressed as a percentile (*M, P*_25_, and *P*_75_) and were compared using a two-sided Mann-Whitney U test between two independent groups, as well as a Wilcoxon signed test between two paired groups. Ranked data were expressed as numbers and percentages (*n*, %), and were compared by a two-sided Mann-Whitney U test for two independent groups, and a two-sided Friedman test for more than two related groups. All above statistical analyses were performed using Microsoft Office Excel 2000 (Microsoft Corporation, Redmond, Washington, the Unite State) and SPSS 23.0 (International Business Machines Corporation, Armonk, New York, the Unite State). The sample size was estimated using the Power and Sample Size online calculator (http://www.powerandsamplesize.com). A *p* < 0.05 was considered statistically significant.

### Ethic

This study was approved by the ethics committee of the Children's Hospital, School of Medicine, Zhejiang University (2021-IRB-183). All residents have read the written notification of the study and given their oral informed consent prior to participation.

## Results

### Design of a Simulation Course about Acute Fulminant Myocarditis in Children

**Figure 1:**
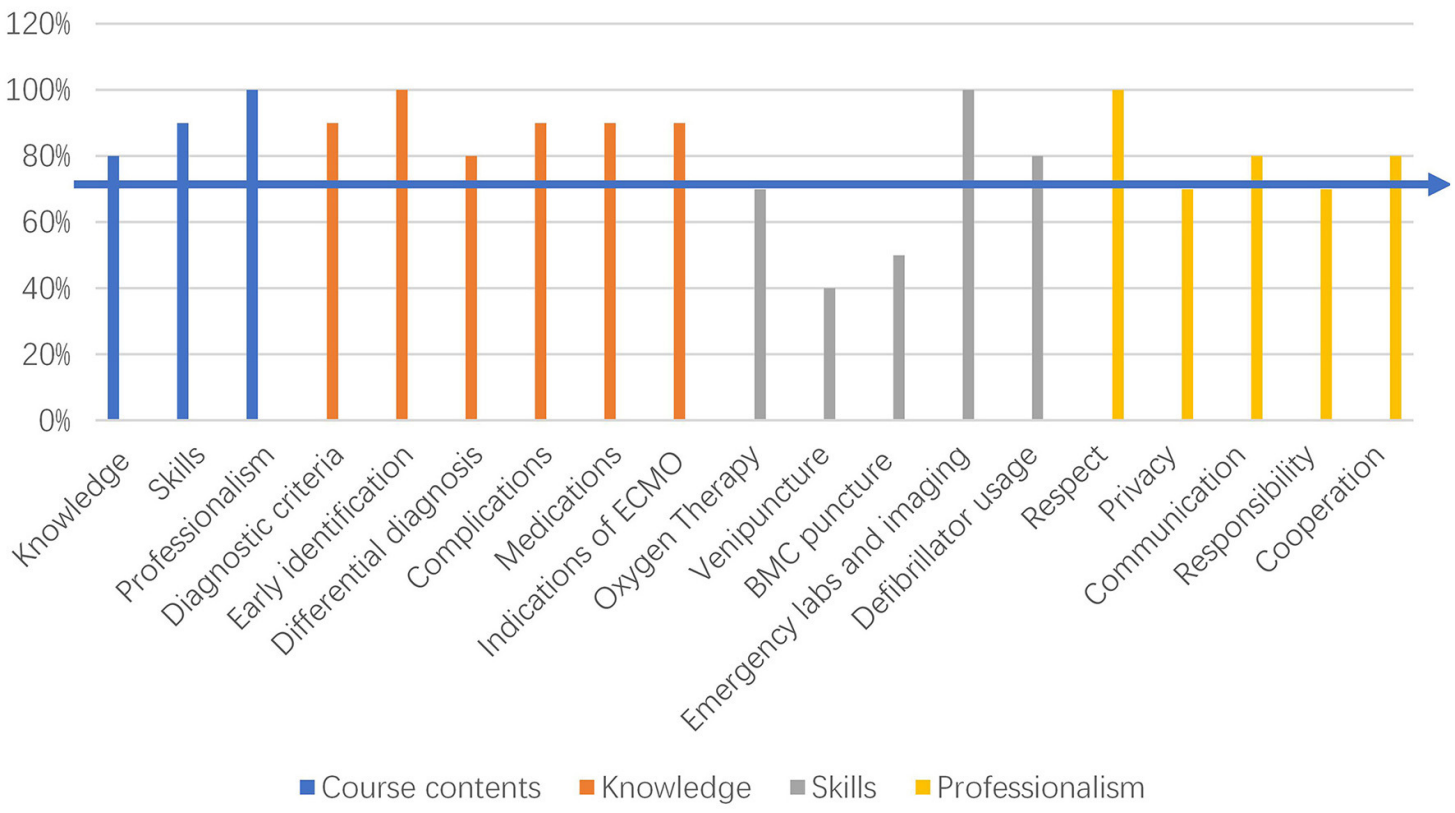
Survey results of the simulation course about acute fulminant myocarditis in children.

**Figure 2:**
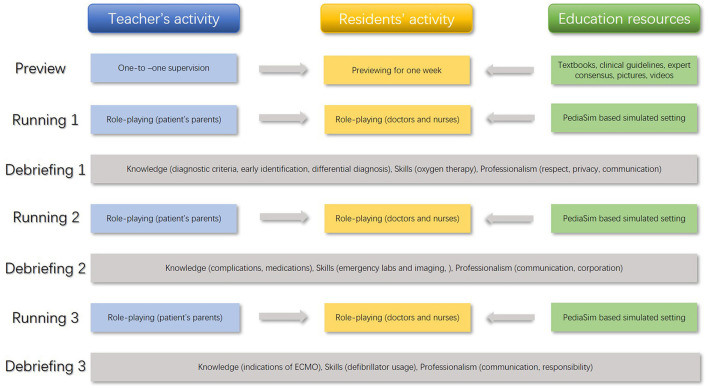
Teaching schedule of the novel simulation course with the segmented model (acute fulminant myocarditis in children).

The mean age of the 10 third-grade pediatric cardiovascular residents was 25.70 ± 0.82 years old and the ratio of male to female was 1:9. All of them reported to expect a simulation curriculum, 60% had heard about a simulation curriculum before, and 40% had participated in a simulation curriculum through other ways. Survey results of the simulation course about AFM in children is shown in Figure 1. Knowledge (diagnostic criteria, early identification, differential diagnosis, complications, medications, and indications for ECMO), skills (oxygen therapy, emergency labs and imaging, defibrillator usage), and professionalism (respect, privacy, communication, responsibility, and cooperation) were selected as the content of the course according to the survey. The details of the teaching schedule of the novel simulation course with the segmented model are shown in Figure 2.

Figure 1 shows the residents' demands for each specific type of course content. The horizontal arrow represents the 70% level, and any content above the 70% level was selected for the course. ECMO, Extracorporeal Membrane Oxygenation; BMC, Bone Marrow Cavity.

Figure 2 shows the teaching schedule of the novel simulation course, including a preview and three cycles of running and debriefing. The content marked in blue represents the teachers' activities, yellow represents the residents' activities, green represents education resources, and gray represents the specific teaching content in each segment. All teachers' activities and education resources served the residents. ECMO, Extracorporeal Membrane Oxygenation.

### Simulation Course Practice

A total of 55 residents completed the whole simulation course practice, including 30 in the experimental group (6 teams) and 25 in the control group (5 teams). Among them, 23 residents in the experimental group and 21 residents in the control group also completed the entire knowledge, skills, and professionalism assessment and satisfaction feedback. Of the 44 residents, the male to female ratios of residents in the experimental and control groups were 0.35:1 and 0.316:1, respectively, and there was no significant difference between the two groups (χ^2^ = 0.03, *p* = 0.570). The time distribution of the two groups is shown in Table 1.

**Table 1:** Time distribution of the experimental and control groups.

	**Control group (min)**	**Experimental group (min)**
**Pre-course test**	10	10
**Introduction**	5	5
**Case practice**		
Running 1	24	8
Debriefing 1	24	8
Running 2	0	8
Debriefing 2	0	8
Running 3	0	8
Debriefing 3	0	8
Post-course test	15	15

### Effectiveness Evaluation

#### Residents' Knowledge Assessment Results

The mean pre-course knowledge assessment score of the residents in the experimental group was 53.04 ± 17.69, while the mean score in the control group was 55.24 ± 28.92, and there was no significant difference between the two groups (*t* = −.0300, *p* = 0.766). The mean post-course knowledge assessment score of the residents in the experimental group was 82.61 ± 17.38, while the mean score in the control group was 50.48 ± 18.57, which was significantly lower than that in the experimental group (*t* = -5.93, *p* < 0.01). The mean post-course knowledge assessment score of the residents was significantly higher than that of the pre-course in the experimental group (*t* = −6.314, *p* < 0.001), while the mean post-course score of the residents was not significantly different from that of pre-course in the control group (*t* = 0.653, *p* = 0.521).

#### Residents' Skills Assessment Results

The mean skills score of residents in the experimental group was 84.17 ± 6.01, which was significantly higher than that in the control group (54.50 ± 5.72, *t* = 8.365, *p* < 0.001).

#### Residents' Professionalism Assessment Results

The residents' professionalism assessment included respect for patients and families, protection of patient privacy, communication with others, responsibility for patients, and cooperation with the team. The professionalism assessment of residents in the experimental group was higher than that in the control group in all aspects (Table 2).

**Table 2:** Residents' professionalism assessment in the experimental and control groups.

**Indicators**	**Control group (*****n*** **=** **5)** **(*****n*****, %)**			**Experimental group (*****n*** **=** **6)** **(*****n*****, %)**			***p***	***Z***
	**1**	**2**	**3**	**1**	**2**	**3**		
Respect	3 (60.0)	2 (40.0)	0 (0.0)	0 (0.0)	0 (0.0)	6 (100.0)	0.004	-3.028
Privacy	2 (40.0)	3 (60.0)	0 (0.0)	0 (0.0)	1 (16.7)	5 (83.3)	0.009	-2.659
Communication	4 (80.0)	1 (20.0)	0 (0.0)	0 (0.0)	1 (16.7)	5 (83.3)	0.004	-2.856
Responsibility	2 (40.0)	3 (60.0)	0 (0.0)	0 (0.0)	0 (0.0)	6 (100)	0.004	-3.028
Cooperation	3 (60.0)	2 (40.0)	0 (0.0)	0 (0.0)	3 (50.0)	3 (50.0)	0.030	-2.345

*1, 2, and 3 represent dissatisfied, average, and satisfied, respectively*.

#### Residents' Satisfaction Feedback

##### Self-Performance Evaluation

The self-performance evaluation showed that residents in the experimental group were significantly more confident regarding their knowledge mastery than those in the control group (*p* = 0.039), and there was no significant difference between the two groups in other aspects (*p* > 0.05) (Table 3).

**Table 3:** Evaluation of the residents' self-performance.

**Indicators**	**Control group (*****n*** **=** **21)** **(*****n*****, %)**					**Experimental group (*****n*** **=** **23)** **(*****n*****, %)**					***p***	***Z***
	**1**	**2**	**3**	**4**	**5**	**1**	**2**	**3**	**4**	**5**		
Knowledge mastery	0 (0.0)	1 (4.8)	6 (28.6)	6 (28.6)	8 (38.1)	0 (0.0)	0 (0.0)	1 (4.3)	8 (34.8)	14 (60.9)	0.039	-2.065
Skills development	0 (0.0)	0 (0.0)	0 (0.0)	8 (38.1)	13 (61.9)	0 (0.0)	0 (0.0)	0 (0.0)	9 (39.1)	14 (60.9)	0.944	-0.070
Professionalism training	0 (0.0)	0 (0.0)	0 (0.0)	5 (23.8)	16 (76.2)	0 (0.0)	0 (0.0)	0 (0.0)	8 (34.8)	15 (65.2)	0.431	-0.788
Careful thinking	0 (0.0)	0 (0.0)	3 (14.3)	6 (28.6)	12 (57.1)	0 (0.0)	0 (0.0)	0 (0.0)	12 (52.2)	11 (47.8)	0.905	-0.119
Active suggestions	0 (0.0)	0 (0.0)	3 (14.3)	5 (23.8)	13 (61.9)	0 (0.0)	0 (0.0)	0 (0.0)	10 (43.5)	13 (56.5)	0.957	-0.054
Proactive questioning	0 (0.0)	0 (0.0)	0 (0.0)	7 (33.3)	14 (66.7)	0 (0.0)	0 (0.0)	0 (0.0)	11 (47.8)	12 (52.2)	0.334	-0.965

*1, 2, 3, 4, and 5 represent very dissatisfied, dissatisfied, average, satisfied, and very satisfied, respectively*.

##### Teacher's Performance Evaluation

The teacher's performance evaluation included simulation design, simulation running, and debriefings. No significant differences between the two groups were found, as shown in Tables 4A–C.

**Table 4A:** Evaluation of the teacher's performance (simulation design).

**Indicators**	**Control group (*****n*** **=** **21)** **(*****n*****, %)**					**Experimental group (*****n*** **=** **23)** **(*****n*****, %)**					***p***	***Z***
	**1**	**2**	**3**	**4**	**5**	**1**	**2**	**3**	**4**	**5**		
Adequate preparation	0 (0.0)	0 (0.0)	0 (0.0)	6 (28.6)	15 (71.4)	0 (0.0)	0 (0.0)	0 (0.0)	5 (21.7)	18 (78.3)	0.605	-0.517
Clear objectives	0 (0.0)	0 (0.0)	0 (0.0)	5 (23.8)	16 (76.2)	0 (0.0)	0 (0.0)	0 (0.0)	7 (30.4)	16 (69.6)	0.626	-0.487
Sufficient information	0 (0.0)	0 (0.0)	0 (0.0)	5 (23.8)	16 (76.2)	0 (0.0)	0 (0.0)	0 (0.0)	8 (34.8)	15 (65.2)	0.431	-0.788
Appropriate threads	0 (0.0)	0 (0.0)	0 (0.0)	4 (19.0)	17 (81.0)	0 (0.0)	0 (0.0)	0 (0.0)	6 (26.1)	17 (73.9)	0.582	-0.550

*1, 2, 3, 4, and 5 represent very dissatisfied, dissatisfied, average, satisfied, and very satisfied, respectively*.

**Table 4B:** Evaluation of the teacher's performance (simulation running).

**Indicators**	**Control group (*****n*** **=** **21)** **(*****n*****, %)**					**Experimental group (*****n*** **=** **23)** **(*****n*****, %)**					***p***	***Z***
	**1**	**2**	**3**	**4**	**5**	**1**	**2**	**3**	**4**	**5**		
Identify needs	0 (0.0)	0 (0.0)	0 (0.0)	5 (23.8)	16 (76.2)	0 (0.0)	0 (0.0)	0 (0.0)	7 (30.4)	16 (69.6)	0.626	-0.487
Provide support	0 (0.0)	0 (0.0)	0 (0.0)	5 (23.8)	16 (76.2)	0 (0.0)	0 (0.0)	0 (0.0)	5 (21.7)	18 (78.3)	0.871	-0.162
Enhance capabilities	0 (0.0)	0 (0.0)	0 (0.0)	4 (19.0)	17 (81.0)	0 (0.0)	0 (0.0)	0 (0.0)	5 (21.7)	18 (78.3)	0.827	-0.219
Explore methods	0 (0.0)	0 (0.0)	1 (4.8)	5 (23.8)	15 (71.4)	0 (0.0)	0 (0.0)	0 (0.0)	5 (21.7)	18 (78.3)	0.553	-0.593
Provide opportunities	0 (0.0)	0 (0.0)	0 (0.0)	4 (19.0)	17 (81.0)	0 (0.0)	0 (0.0)	0 (0.0)	5 (21.7)	18 (78.3)	0.827	-0.219

*1, 2, 3, 4, and 5 represent very dissatisfied, dissatisfied, average, satisfied, and very satisfied, respectively*.

**Table 4C:** Evaluation of the teacher's performance (simulation debriefing).

**Indicators**	**Control group (*****n*** **=** **21)** **(*****n*****, %)**					**Experimental group (*****n*** **=** **23)** **(*****n*****, %)**					***p***	***Z***
	**1**	**2**	**3**	**4**	**5**	**1**	**2**	**3**	**4**	**5**		
Organization	0 (0.0)	0 (0.0)	0 (0.0)	5 (23.8)	16 (76.2)	0 (0.0)	0 (0.0)	0 (0.0)	3 (13.0)	20 (87.0)	0.361	-0.914
Timeliness	0 (0.0)	0 (0.0)	0 (0.0)	4 (19.0)	17 (81.0)	0 (0.0)	0 (0.0)	0 (0.0)	3 (13.0)	20 (87.0)	0.591	-0.538
Inspiration	0 (0.0)	0 (0.0)	0 (0.0)	5 (23.8)	16 (76.2)	0 (0.0)	0 (0.0)	0 (0.0)	4 (17.4)	19 (82.6)	0.602	-0.521
Authenticity	0 (0.0)	0 (0.0)	1 (4.8)	6 (28.6)	14 (66.7)	0 (0.0)	0 (0.0)	0 (0.0)	6 (26.1)	17 (73.9)	0.544	-0.607

*1, 2, 3, 4, and 5 represent very dissatisfied, dissatisfied, average, satisfied, and very satisfied, respectively*.

## Discussion

Although there is still a long way to go in the training of cardiovascular pediatricians, particularly in developing countries such as China, we have been working hard to cultivate qualified pediatricians in three areas: knowledge, clinical skills, and professionalism (14). This is necessary to provide qualified patient care. The ultimate goal of this education is to improve the health statuses of the patients and the community (14). There are common obstacles among some pediatric cardiovascular residents regarding the retention of knowledge, application of clinical skills, and embodiment of professionalism, which brings great challenges to the pediatric cardiovascular residents' training program. The ideal teaching mode should transfer knowledge, skills, and professionalism in the process of medical education in order to fully develop these aspects. The emergence of SBME has brought a new dawn, but the best teaching model is still being explored (3). We tried a novel simulation curriculum with a segmented model, hoping to improve the teaching effectiveness.

First, qualified pediatric cardiovascular doctors must have rich professional knowledge and proficient clinical skills. For example, to manage children with AFM well, pediatric cardiovascular doctors need to master the disease's incidence rate, etiology, clinical features, management, and prognosis. Our course focused on the knowledge related to the diagnosis and therapy of AFM and the related clinical skills. There were significant differences in the focus areas of the three segments. At the knowledge level, the first segment focused on the early recognition of AFM in children, the second segment focused on the preliminary emergency management, and the third segment focused on the indications for ECMO. At the skill level, the first segment focused on the oxygen therapy strategy, the second segment focused on various emergency labs and imaging, and the third segment focused on the use of defibrillators. SBME is a powerful tool for acquiring knowledge and improving skills, and previous studies have shown its ability in the training of pediatricians (15, 16). In our course, we organically integrated these contents into different segments and delivered focused knowledge and skills training in each segment. From the teacher's point of view, the simulation design was more focused, the simulation running was more purposeful, and the debriefing was timelier. From the residents' point of view, the training was shorter, there was less consent, and quicker feedback was also preferable. The design was in line with the teaching principle of moving along step-by-step. Although the content, teachers, pre-course preview were the same, the knowledge and skill assessment outcomes of the two groups were significantly different. The gap in the knowledge and skill scores between the two groups was as high as 30 points, which was very surprising.

Secondly, professionalism is necessary for pediatric cardiovascular specialists and has attracted more and more attention. Courses that focus only on diseases are no longer suitable for medical education. The American Association of Medical Colleges has established a team of experts to address the challenges in medical education as part of the ongoing Medical School Objectives Project (17). The American Academy of Pediatrics has also recently issued professional quality standards for pediatricians (18). Pediatric cardiovascular patients have unique characteristics, such as poor self-expression, low emotional control, intolerance to disease, non-compliance with examination and treatment, and strong dependency on their parents. The family members of the affected children are prone to be anxious and doubt the medical staff. Many studies have shown the effectiveness of health communication interventions in improving doctor-patient/parent relationships and reducing non-compliance. The benefits of doctor-patient communication may exceed these short-term outcomes, as demonstrated by the cases of other adult chronic diseases (19). In addition, the condition of pediatric cardiovascular patients can change rapidly, so the demand for doctor professionalism, including responsibility, teamwork, and crisis resource management, is also very prominent. However, for a long time, courses on professionalism have been rather empty and boring. Recently, SBME has been reported to have been applied to improve professionalism, but the effect has not been confirmed (20). Our study found that the segmented simulation model was helpful for the professionalism training of pediatricians. In the teaching objectives of each segment, we added professionalism training (including teamwork, communication, and so on). Based on the importance of repeated stimulation (cycles of running and debriefing) for the retention of residents' memory and the cultivation of professionalism awareness, the residents' professionalism significantly improved after multiple segments of training. We found that compared with the control group, residents in the experimental group significantly improved in terms of respecting their patients, protecting patient privacy, and communication.

Residents about to enter clinical work have anxiety related to it. In the beginning, residents often lack sufficient abilities in history recording, physical examination, and diagnostic skills, resulting in a lack of confidence in their performers. They often suffer from psychological problems (stress and anxiety) due to the responsibility they have for patients. Significant stress, high anxiety, and low self-confidence increase the possibility of errors and, thus, harm the success of clinical practice (21). It is necessary to improve the ability of those engaging in patient care through various clinical experiences in a safe environment. Residents with rich medical experience showed confidence in patient care, while residents with high confidence also showed improvement in their clinical skills. In other words, confidence increases as they gain experiences (22). For residents who have limited opportunities to face real patients, it is necessary to provide an environment similar to the real clinical situation to help them better prepare themselves for clinical practice (22). This situation is particularly prominent among young pediatric cardiovascular residents because the severity of the cardiovascular disease is always higher than other diseases. SBME provides an environment very similar to the clinical setting so that students can experience the actual clinical management. The human patient simulators in our study are new and effective teaching models, which have been widely used in medical education (3). Human patient simulators allow residents to experience low-frequency or high-risk scenarios safely and improve their knowledge, skills, and professionalism (23). In particular, with the spread of Corona Virus Disease 2019, the number of hospitalized children has decreased rapidly, and the opportunity for medical residents has decreased as well. In this case, human patient simulators can be actively used as an alternative method to improve the confidence of residents. In addition, anxiety occurring under specific circumstances or stimuli can be reduced through repeated contact (24). Some studies have found that high-fidelity simulation experience, especially repeated contact simulation, can decrease the anxiety and self-confidence of medical students (22, 25). In this study, we found that the residents' self-confidence in mastering knowledge in the experimental group was better than that in the control group. Residents received feedback, repeated similar but more difficult tasks, and saw their progress, which further improved feelings of competence and made learners more welcoming to feedback (5).

Our study proved the feasibility and effectiveness of a simulation curriculum with the segmented model for the first time. The knowledge, skill, and professionalism performance of the residents were significantly improved by applying the novel model. In addition, it also increased the residents' self-confidence. In the analysis of the satisfaction with the teachers, the two groups were comparable in simulation design, the running, and the debriefings. This indicated that the enhanced effectiveness of the experimental group came from the multiple-segmented model of simulation itself rather than from the teacher's skills. The design of the case went deeper step-by-step, which not only aroused residents' interest and self-confidence but also made full use of the two SBME metrics, thereby achieving greater teaching effectiveness.

Our study has some limitations. First, our research lacks the comparison of the patients' improvement after the training of residents through the traditional and novel models, respectively. We will follow up in the future with further studies focused on this area, which will provide more information about the teaching effectiveness of the novel model. Second, the follow-up period is short and does not fully reflect the effect of the long-term retention of knowledge and skills through the novel simulation teaching model. We will extend the follow-up period in our future work to further analyze it. In addition, although the novel simulation curriculum received positive feedback, the findings were only based on one teaching practice. We will, thus, conduct more teaching practices with more participants in future studies to support the findings reported in this study. Finally, although it appeared that the professionalism performance of residents improved based on the qualitative results, there was a lack of quantitative assessment. We will try to develop a more scientific evaluation system in the future.

In conclusion, the simulation curriculum with the novel segmented model could improve the knowledge, skills, and professionalism performance of pediatric cardiovascular residents and enhance their self-confidence. The course received positive feedback, which is worthy of further promotion and application. In future research, we plan to apply this novel simulation model to other pediatric curricula and develop a series of coherent curriculum systems. We will promote it among more pediatric teachers and students, hoping to improve the service quality of pediatric medical workers. Second, after the training, we will regularly follow up on the clinical performance of residents, the improvement and satisfaction of patients, and give feedback to residents and teachers in a timely manner. In addition, we will optimize the assessment system, including a multidimensional evaluation of resident performance, hoping to reflect the teaching effect of curricula and the learning effect of residents through a more reliable method.

## Data Availability Statement

The original contributions presented in the study are included in the article/supplementary material, further inquiries can be directed to the corresponding author.

## Ethics Statement

The studies involving human participants were reviewed and approved by the Ethics Committee of the Children's Hospital, School of Medicine, Zhejiang University. Written informed consent for participation was not required for this study in accordance with the national legislation and the institutional requirements.

## Author Contributions

YY collected the data, guided the simulation course practice, and wrote the draft. L-FT, C-ZH, and J-HM performed the statistical analysis and revised the manuscript. Y-XH designed the study and critically reviewed the manuscript for important intellectual content. All authors approved the final manuscript as submitted and agreed to be accountable for all aspects of the work.

## Funding

The Major Project of New Generation Artificial Intelligence, Scientific and Technological Innovation 2030: Application Research of Virtual Standard Pediatric Patient Model (Ministry of Science and Technology of the People's Republic of China) (2021ZD0113505). The Second Batch of Industry-University-Education Collaboration Projects: Exploration and Practice of Virtual Reality Augmented Technology in Pediatric Intubation Education (Ministry of Education of the People's Republic of China) (202102177013). The General Project: Development and Application of Advanced Curriculum of Pediatric Emergency and Critical Care Based on High-Fidelity Simulators (Zhejiang Provincial Health Commission) (2021427236).

## Conflict of Interest

The authors declare that the research was conducted in the absence of any commercial or financial relationships that could be construed as a potential conflict of interest.

## Publisher's Note

All claims expressed in this article are solely those of the authors and do not necessarily represent those of their affiliated organizations, or those of the publisher, the editors and the reviewers. Any product that may be evaluated in this article, or claim that may be made by its manufacturer, is not guaranteed or endorsed by the publisher.
